# Glycemic index, glycemic load, and lung cancer risk: A meta-analysis of cohort and case-control studies

**DOI:** 10.1371/journal.pone.0273943

**Published:** 2022-09-01

**Authors:** Hongzhen Du, Tianfeng Zhang, Xuning Lu, Meicui Chen, Xiaoling Li, Zengning Li

**Affiliations:** 1 Department of Nutrition, The First Hospital of Hebei Medical University, Shijiazhuang, Hebei, China; 2 Key Laboratory of Nutrition and Health of Hebei Province, Shijiazhuang, China; University Putra Malaysia (2005-2018), MALAYSIA

## Abstract

**Objective:**

Glycemic index (GI) or glycemic load (GL) has been investigated in the field of cancer research for several years. However, the relationship between GI or GL and lung cancer risk remains inconsistent. Therefore, this study aimed to summarize previous findings on this relationship.

**Methods:**

PubMed, Embase, Scopus, Web of Science databases, and Cochrane Library were searched by July 2021. This review was conducted in accordance with the PRISMA guidelines. A fixed or random-effects model was adopted for meta-analysis to compute the pooled relative risks (RR) and their corresponding 95% confidence intervals (CIs). Subgroup analyses, sensitivity analyses, and publication bias analyses were also performed.

**Results:**

In total, nine articles were included, with four case-control studies and five cohort studies, including 17,019 cases and 786,479 controls. After merging the studies, pooled multivariable RRs of lung cancer based on the highest versus the lowest intake were 1.14 (95%CI: 1.03–1.26) and 0.93 (95%CI: 0.84–1.02) for GI and GL. Results persisted in most stratifications after stratifying by potential confounders in the relationship between GI and lung cancer risk. There was a non-linear dose response relation for GI with lung caner risk.

**Conclusion:**

GI typically has a positive relationship with lung cancer risk. However, no associations between GL and lung cancer risk were observed based on current evidence, suggesting that this issue should be studied and verified further to substantiate these findings.

## Introduction

Lung cancer exhibits the fastest growth in morbidity and mortality and a large threat to health worldwide. According to the global cancer statistics in 2020 [1], the number of new cases and deaths of lung cancer are the second and first among all cancers, respectively. Lung cancer is the first high-incidence cancer among men in 36 countries and the first in the spectrum of cancer incidence and death in China [1]. However, the overall 5-year survival rate is still < 20% [2]. Behavioral risk factors of lung cancer contain tobacco smoking and air pollution. Several studies have indicated the association of diet with lung cancer risk [3].

Evidence suggests that dietary habits play an important role in altering lung cancer risk. Studies have found that fruits, vegetables, fish, and dairy products may be associated with lung cancer risk [4]. Moreover, investigations have focused on diet, carbohydrates, and their impacts on human health for years [5, 6]. In addition, high carbohydrate intake changes the insulin-like growth factor (IGF) I pathway [7], producing oxidative stress and promoting cell proliferation, leading to cancer [8]. Prior studies have elucidated that IGF-1 is elevated in patients with lung cancer, suggesting that elevated circulating insulin may play a role in lung cancer [9]. In a cohort of 2.3 million adults, the lung cancer prevalence in the diabetes mellitus (DM) population and in those with incident diabetes was 8.7% and 9.3%, respectively, while the prevalence of free diabetes was 8.2% [10]. However, a recent meta-analysis shows no correlation between DM and lung cancer risk in men, but a significant correlation was observed in women [11]. Therefore, the abnormal glucose metabolism and hyperinsulinemia role in lung cancer etiology deserve further attention.

The ability of carbohydrates to affect blood sugar and insulin concentrations varies widely, depending largely on the amount and type of carbohydrates consumed. In 1981, the glycemic index (GI) was developed to classify carbohydrate-rich foods based on their postprandial glycemic effect [12]. The GI is defined as the incremental area under the glycemic response curve after consuming 50 g of available carbohydrates in food [12]. GL is a measure of the overall glucose response to food and is calculated by multiplying the grams of food consumed by the GI of the food. Although the impact of GI and GL on human health have been studied for a long time, there lacks a consensus [13]. An overview suggested that high GI and GL can increase cancer risk, which fueled an ongoing debate regarding their relevance to cancer incidences [14]. However, previous publications of different cancers showed discrepant results. For example, some studies reported that GI and GL might be associated with the risk of bladder, breast, gastric, colorectal, and ovarian cancers; however, no significant association for pancreatic cancers [15–20]. Two meta-analyses have examined the relationship between GI or GL and lung cancer risk [21, 22]; however, these articles have limitations, such as insufficient included studies, unexplained heterogeneity sources, and excluded case-controlled studies. Increasing attention has been given to evidence-based medicine, thereby warranting the collation of previous data and summary of results. Therefore, in this study, a systematic review and meta-analysis of all eligible studies was conducted to illustrate the association of GI or GL with lung cancer risk.

## Materials and methods

### Search strategy

This study was conducted in accordance with the Meta-Analysis of Observational Studies in Epidemiology guidelines [23]. Two independent investigators (HZD and TFZ) searched all the studies included in the PubMed, Cochrane, Embase, Scopus, and Web of Science electronic databases before July 2021. Search strategy included MeSH and free terms: “Lung Neoplasms” combined with “Glycemic index” or “Glycemic Load.” In addition, articles retrieved from references and other reviews were examined to avoid missing relevant studies. The titles and abstracts of all searched papers were checked for inclusion eligibility, resolving disagreements by discussion.

### Selection of articles

Studies were included based on the following criteria: (1) the types of studies were case-control, nested case-control, cohort, and case-cohort studies similar to follow-up studies of randomized clinical trial design; (2) investigation of the relationship between GI or GL and lung carcinoma risk; (3) the primary result was lung cancer incidence; and (4) relative risk (odds or hazard ratios) of lung cancer, and the 95% CI, or sufficient data to fully estimate these values. For dose response meta-analysis, a quantitative measure of GI or GL at least three quantitative categories and the total number of cases and participants/person-years or non-cases had to be provided. In the case of duplicate studies, only the study that included the most lung cancer cases or the latest publication was included.

### Data extraction

Two independent investigators (HZD and TFZ) obtained the following data from eligible research: author’s name, issuing time, area, sex, study design, cases and control number and source, dietary evaluation methods, confounders, and statistics.

### Study quality assessment

The same two researchers (HZD and TFZ) utilized the Newcastle-Ottawa Scale(NOS, http://www.ohri.ca/programs/clinical_epidemiology/oxford.asp) to assess the methodological quality of each included study [24]. Any inconsistent issues were resolved by discussion and was evaluated based on the following aspects: patient selection (four points), comparability (two points), and outcome assessment (three points). Consequently, the interval of quality scores was 0 (lowest) to 9 (highest), and studies greater than or equal to 6 were considered high quality.

### Statistical methods

Our main analyses were focused on the association between GI or GL and lung cancer risk. The various measures of RR (i.e., odds, rate, and risk ratios) were unanimously considered. In addition, the fully adjusted RRs with their 95% CIs were extracted and transformed into log RR. Greenland’s formula was used to calculate corresponding variance [25]. Heterogeneity among studies was explored using *Q* and *I*^*2*^ statistics. When the *P*-value for the *Q* statistic was < 0.1 or the *I*^*2*^ was > 50%, the studies indicated statistically significant heterogeneity [26]. In addition, when heterogeneity was detected, then a random effects model was used for meta-analysis otherwise a fixed-effects model was utilized. Sub-group analyses were used based on the study characteristics to evaluate the effects of these factors on outcomes. Meta-regression was adopted to assess between-study heterogeneity. Furthermore, we performed the overall risk estimate using sensitivity analysis by successively omitting each study to estimate the specific study effects on the results. Funnel plot, Begg’s rank correlation test and Egger’s regression asymmetry test were used for assessing publication bias. For dose response meta-analysis, we performed generalised least-squares trend estimation (GLST) which was established by Greenland and Longnecker [27]. Potential non-linear associations were explored by using restricted cubic splines [28]. We computed a *P* value for nonlinearity by testing the null hypothesis that the coefficient of the second spline is equal to zero. Statistical analysis was performed by using STATA 12.0 (StataCorp, College Station, TX, USA). A two-sided *P* < 0.05 showed significant differences.

## Results

**Fig 1:**
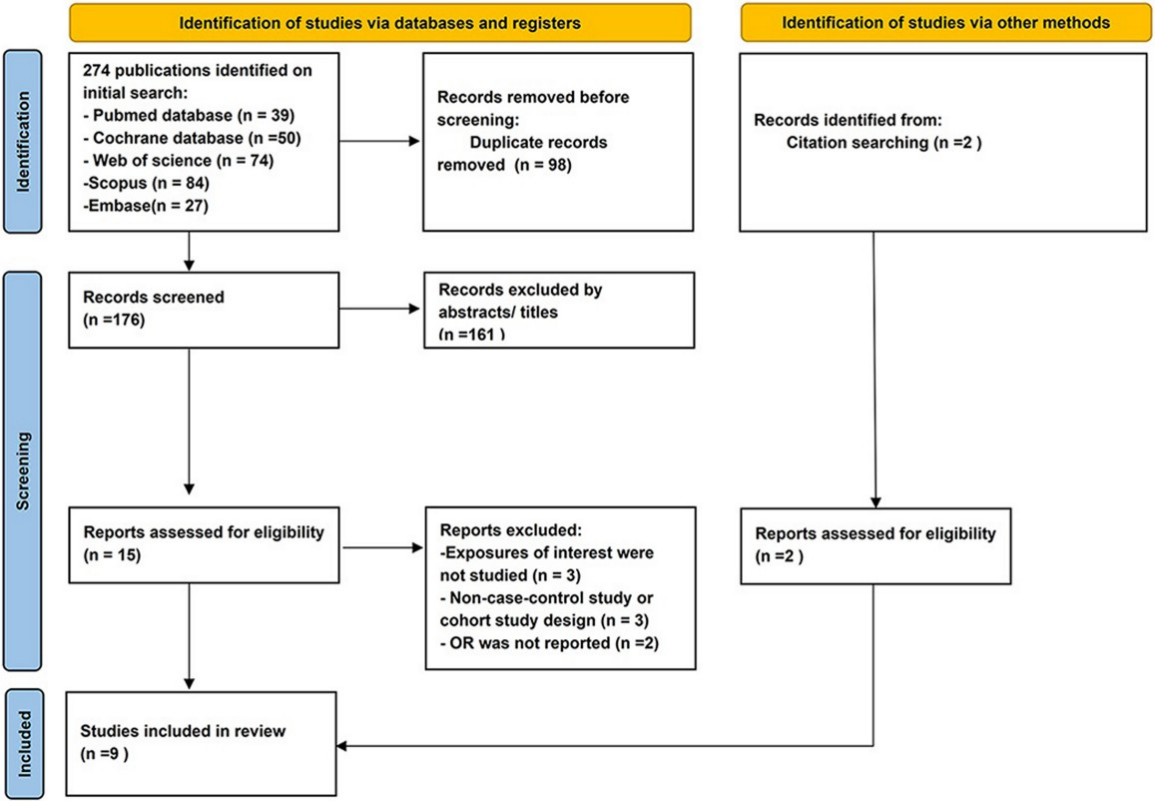
Prisma 2009 flow diagram literature search and study selection. PRISMA diagram showing the different steps of systematic review, starting from literature search to study selection and exclusion. The reasons for exclusion are indicated for each step.

Fig 1 shows a flow chart of the research selection process. During the initial search, 274 articles were identified through PubMed, Embase, Scopus, Web of Science databases, and Cochrane Library, and two additional records were screened through other source (hand-searching references of identified papers). After excluding 98 duplicates from different databases, 176 articles remained for the title and abstract screening. Then, 15 full-text articles were examined. Seven studies were then excluded, and the reasons for exclusion are described in Fig 1. Finally, nine eligible articles were included in the meta-analysis, including five cohort and four case-controlled studies [29–37]. Of these, two cohorts only identified risk estimates divided by sex [29, 35]. Eleven related studies were identified (four case-control and seven cohort studies).

Four studies were conducted from America, two from China, one from Uruguay, one from Italy, and one from Canada. Table 1 shows the basic features of all citations. The meta-analysis consisted of 17,019 cases and 786,479 controls. The sample size ranged from 928 to 183,535, which were 803,498 for GI and 802,570 for GL, and 947 to 3,769 lung cancer cases occurred among the participants. The adjusted major confounding variables included age, sex, education, body mass index, smoking status, total energy intake, family history of lung cancer, and physical activity. In the study quality assessment, the quality characteristics of all included studies were 7–9 points, indicating high-quality reports (Table 2).

**Table 1:** Main characteristics of previous studies examined the association of GI, GL, and lung cancer risk.

Study (Year)	Area	Study Design	Time	Gender	Total	Cases		Control		Dietary assessment method; Reference food for GI/GL	**OR (95% CI) Comparison level (highest vs. lowest)**		Adjustments
											**GI**	**GL**	
De Stefani, 1998	Uruguay	Case control study	1993–1996	F&M	928	463		465		64 items FFQ	2.77 (1.28–5.97)		Age, residence, urban/rural status, education and income
J. Hu, 2012	Canada	Case control study	1994–1997	F&M	8,380	3341		5,039		69 items FFQ	1.04 (0.89–1.23)	0.98 (0.80–1.21)	Smoke
Melkonian, 2016	USA, Houston	Case control study	12 months	F&M	4,318	1905		2,413		National Cancer Institute Health Habits and History Questionnaire	Q5:Q1 1.49 (1.21–1.83)	Q5:Q1 1.16 (0.94–1.42)	Age, sex, education (<12 years, 12–15 years, 16+ years), ethnicity, BMI, and smoking status
Chang, 2020	USA, Los Angeles	Case control study	1999–2004	F&M	1619	593		1026		78 items FFQ	T3:T1 1.62 (1.17–2.25)	T3:T1 1.13 (0.79–1.64)	Age, sex, education (<12 years, 12–16 years, 17+ years), ethnicity, BMI, and smoking
Study, Year	Area	Study Design	Time/follow-up	Gender	Cohort size		Cases		Dietary Assessment Method; Reference Food for GI/GL		RR/ HR (95% CI) Comparison Level (Highest vs. Lowest)		Adjustments
											GI	GL	
George, 2009	USA	Cohort Study	1995–2003	F&M	446,177W: 183,535M: 262,642		Total: 6,057 W: 2,288 M: 3,769		124 items FFQ		Q5:Q1 W: 1.12(0.98–1.27) M: 1.08(0.98–1.20)	Q5:Q1 W: 0.81(0.64–1.03) M: 0.93(0.78–1.11)	Age, race/ethnicity, education, marital status, BMI, family history of any cancer, physical activity, smoking, alcohol consumption, total energy intake, and menopausal hormone therapy use
Sieri, 2017	Italy	Cohort Study	1993/1998–2009/2010	F&M	45,148		307		validated FFQs		Q5:Q1 0.88(0.53–1.46)	Q5:Q1 0.88(0.53–1.46)	sex, education, smoking status, BMI, intake of alcohol, fiber, saturated fat, and non-alcohol energy, and physical activity
Sun, 2018	China, Shanghai	Cohort Study	W:1997.3–2000.5 M:2002.4–2006.6 Follow up until 2013.12.31	F&M	Total: 130,858 W: 71,663 M: 59,195		1312 W:649 M:663		77 items FFQ		Q4: Q1 W: 1.16(0.92–1.47) M: 0.83(0.67–1.03)	Q4: Q1 W: 1.09(0.86–1.37) M: 0.85(0.68–1.05)	Age, energy intake, smoking status (only men), education, income
Shu, 2020	USA	Cohort Study	2002–2016	F&M	55068		947		89 items FFQ		Q5: Q1 1.06(0.86–1.30)	Q5: Q1 0.88(0.71–1.07)	Sex, race, smoking pack-year, education level, annual house- hold income, COPD, asthma, BMI, family history of lung cancer, and recruitment method
Tao, 2021	China, Hong Kong	Cohort Study	1993–2001, 10-year follow-up	F&M	111,002		2094		The Diet History Questionnaire (DHQ) version 1.0 (National Cancer Institute, 2007)		Q4: Q1 1.19(1.05–1.35) W: 1.10(0.91–1.33) M: 1.23(1.04–1.46)	Q4: Q1 0.76(0.65–0.90) W: 0.75(0.58–0.97) M: 0.74(0.60–0.93)	Age, sex, race, family lung cancer history, education level, BMI and smoking intensity

Note: F = female; M = man; FFQ = food frequency questionnaire; COPD = chronic obstructive pulmonary disease; BMI = body mass index

**Table 2:** Methodological quality of studies included in the meta-analysis.

Case-control studies	Selection				Comparability	Exposure			Score
	Case definition	Representativeness	Control selection	Control definition	Control for important factor or additional factor	Ascertainment of exposure	Same method of ascertainment for cases and controls	Non-Response rate	
Eduardo De Stefani, 1998	*		*	*	**	*	*	*	8
J. Hu, 2012	*		*	*	**		*	*	7
Stephanie C Melkonian, 2016	*	*	*	*	**	*	*		7
Chun-Pin Chang, 2020	*	*	*	*	**	*	*		7
Cohort studies	Selection				Comparability	Outcome			Score
	Representativeness of the exposed cohort	Selection of the unexposed cohort	Ascertainment of exposure	Outcome of interest not present at start of study	Control for important factor or additional factor	Outcome assessment	Follow-up long enough for outcome to occur	Adequacy of follow-up of cohorts	
Stephanie Materese George, 2009		*	*	*	**	*	*	0	7
Jiang-Wei Sun, 2018	*	*	*	*	**	*	*	*	9
Sieri,2017	*	*	*		**	*	*		7
Xiang Shu, 2020	*	*	*		**	*	*	*	8
Jun Tao, 2021	*	*	*	*	**	*	*		8

### GI and lung cancer risk

**Fig 2:**
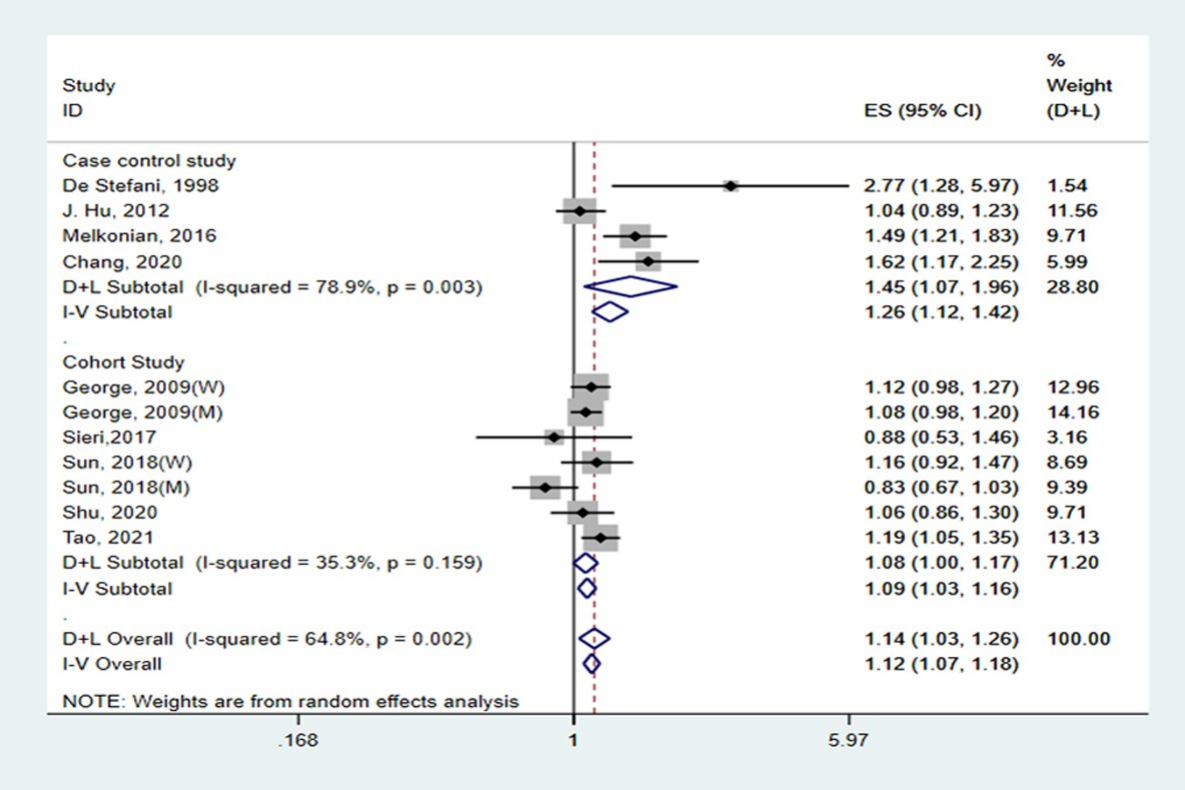
Forest plot showing risk estimates of the association between GI and lung cancer risk.

**Fig 3:**
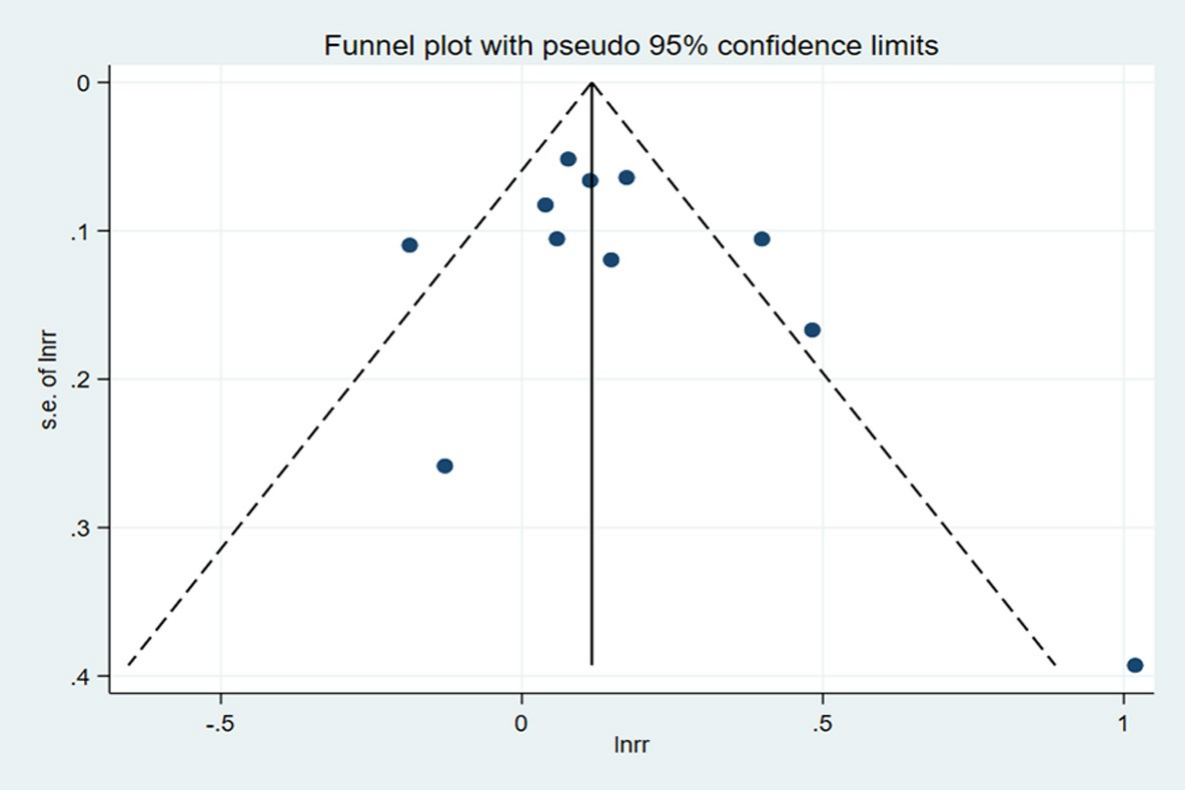
Funnel plot for the association between GI and lung cancer risk.

**Fig 4:**
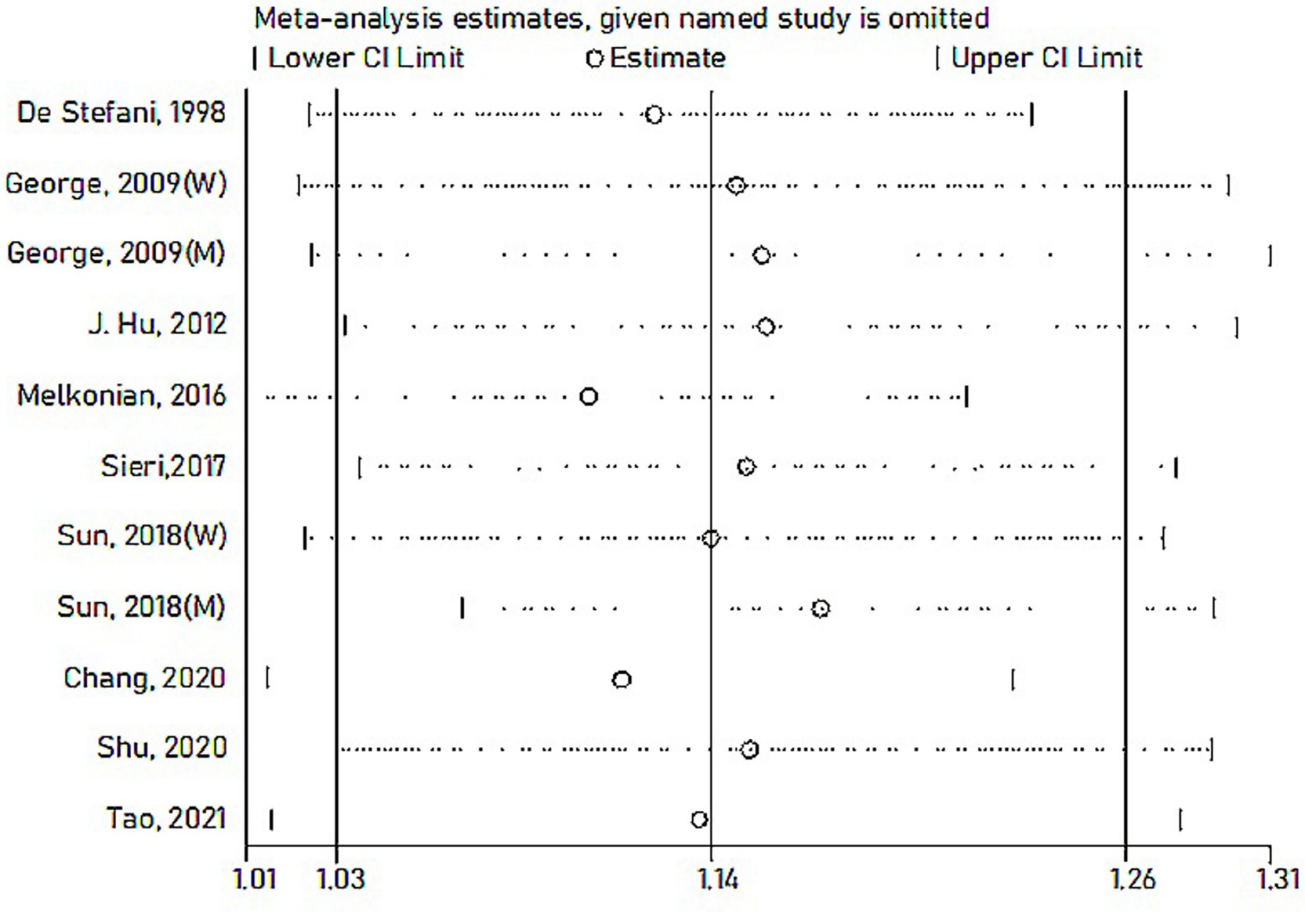
Sensitivity analysis for the association between GI and lung cancer.

**Fig 5:**
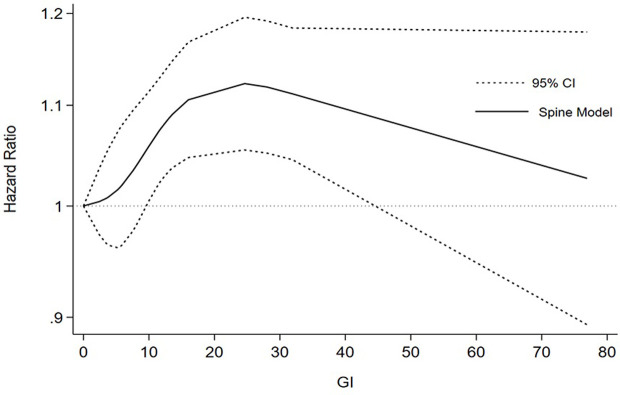
Dose-response association between GI and lung cancer.

Among the nine included studies, four case-control and five cohort studies were included on GI and lung cancer risk. Two cohorts included both men and women [29, 35]. A significant positive relationship between GI and lung cancer risk was found, and the RR summary was 1.14 (1.03–1.26), with evident heterogeneity (*I*^*2*^ = 64.8%; *P-heterogeneity* = 0.002; Fig 2). Therefore, random-effects model estimates were used. There was no evidence of publication bias found in Egger’s test (*P* = 0.361) or Begg’s test (*P* = 0.533). The funnel plot result is presented in Fig 3. The pooled results remained unchanged in this sensitivity analysis (Fig 4). We performed a meta-regression to obtain the potential heterogeneity sources (Table 3). We analyzed the covariables design, region, sex, quality, and energy intake adjustment. However, none of these factors were significantly associated with the difference in GI. In subgroup analyses, the findings were 1.45 (1.07–1.96) and 1.08 (1.00–1.17) in case-controlled and cohort studies, respectively. Six studies on the association between GI and lung cancer were included in the dose-response analysis [30–32, 35–37]. There was an evidence of a curvilinear relationship between the GI and risk of lung cancer risk (*P* for nonlinearity  = 0 .0081). As shown in Fig 5, there was a positive correlation between the dosages of ~ 9.5–44.5 of GI and the lung cancer risk.

**Table 3:** Subgroup analyses of GI and GL and lung cancer risk, high vs. low intake.

Subgroups		N	RR (95% CI)	*P* value	Q statistic	I^2^	*P*-heterogeneity	*P*-interaction
GI		11	1.14 (1.03–1.26)	0.009	28.4	64.80%	0.002	
Design	Case-control	4	1.45 (1.07–1.96)	0.017	14.22	78.90%	0.003	
	Cohort	7	1.08 (1.00–1.17)	0.060	9.27	35.30%	0.159	0.081
Region	America	5	1.21 (1.05–1.38)	0.007	12.48	67.90%	0.014	
	others	6	1.08 (0.91–1.28)	0.377	14.96	66.60%	0.011	0.394
Sex	overall	6	1.08 (0.99–1.18)	0.073	7.70	35.10%	0.174	
	Male	3	1.04 (0.86–1.25)	0.710	6.73	70.30%	0.035	
	Female	3	1.12 (1.02–1.24)	0.021	1.12	0	0.942	0.309
Adjustment for energy intake	Yes	5	1.05 (0.94–1.16)	0.396	6.87	41.80%	0.143	
	No	6	1.27 (1.08–1.49)	0.004	16.38	69.50%	0.006	0.139
GL		10	0.93(0.84–1.02)	0.117	15.59	42.30%	0.076	
Design	Case-control	3	1.08 (0.94–1.23)	0.296	1.36	42.30%	0.506	
	Cohort	7	0.87 (0.80–0.94)	0.003	7.21	16.70%	0.302	0.048

*P*-heterogeneity, heterogeneity within each subgroup; *P*-interaction, heterogeneity between subgroups with meta-regression analysis

### GL and lung cancer risk

**Fig 6:**
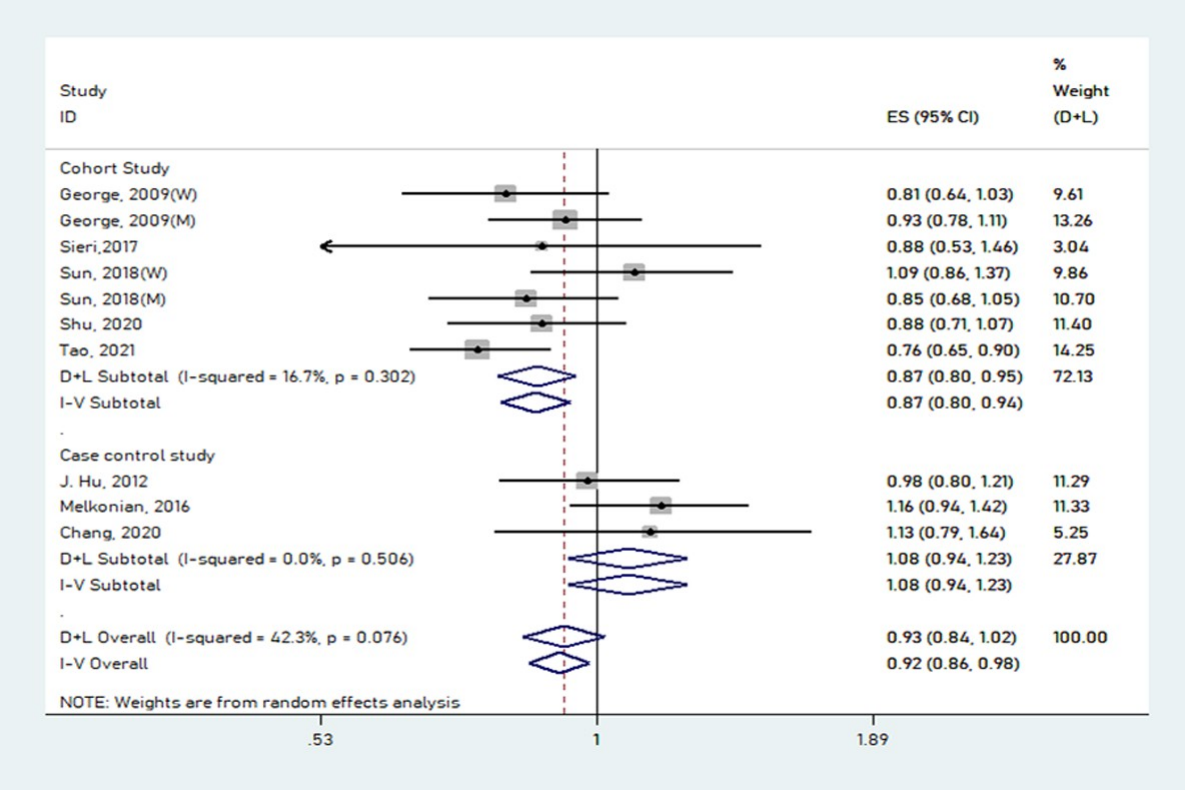
Forest plot showing risk estimates of the association between GI and lung cancer risk.

**Fig 7:**
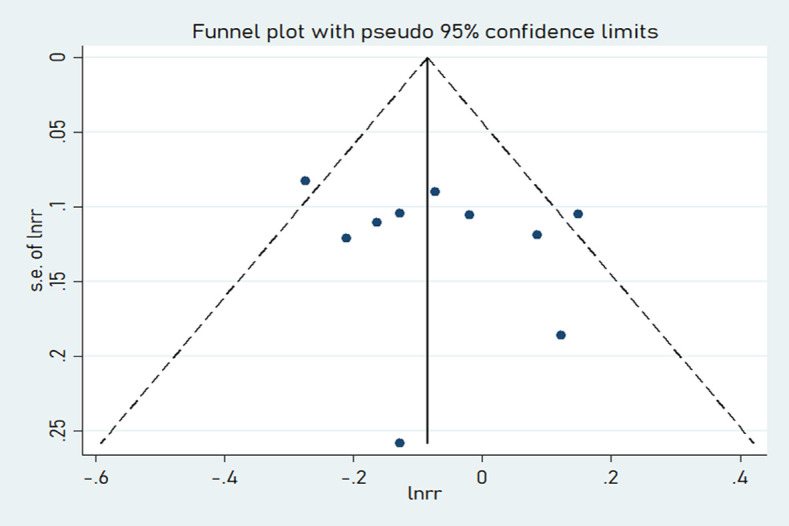
Funnel plot for the association between GL and lung cancer risk.

**Fig 8:**
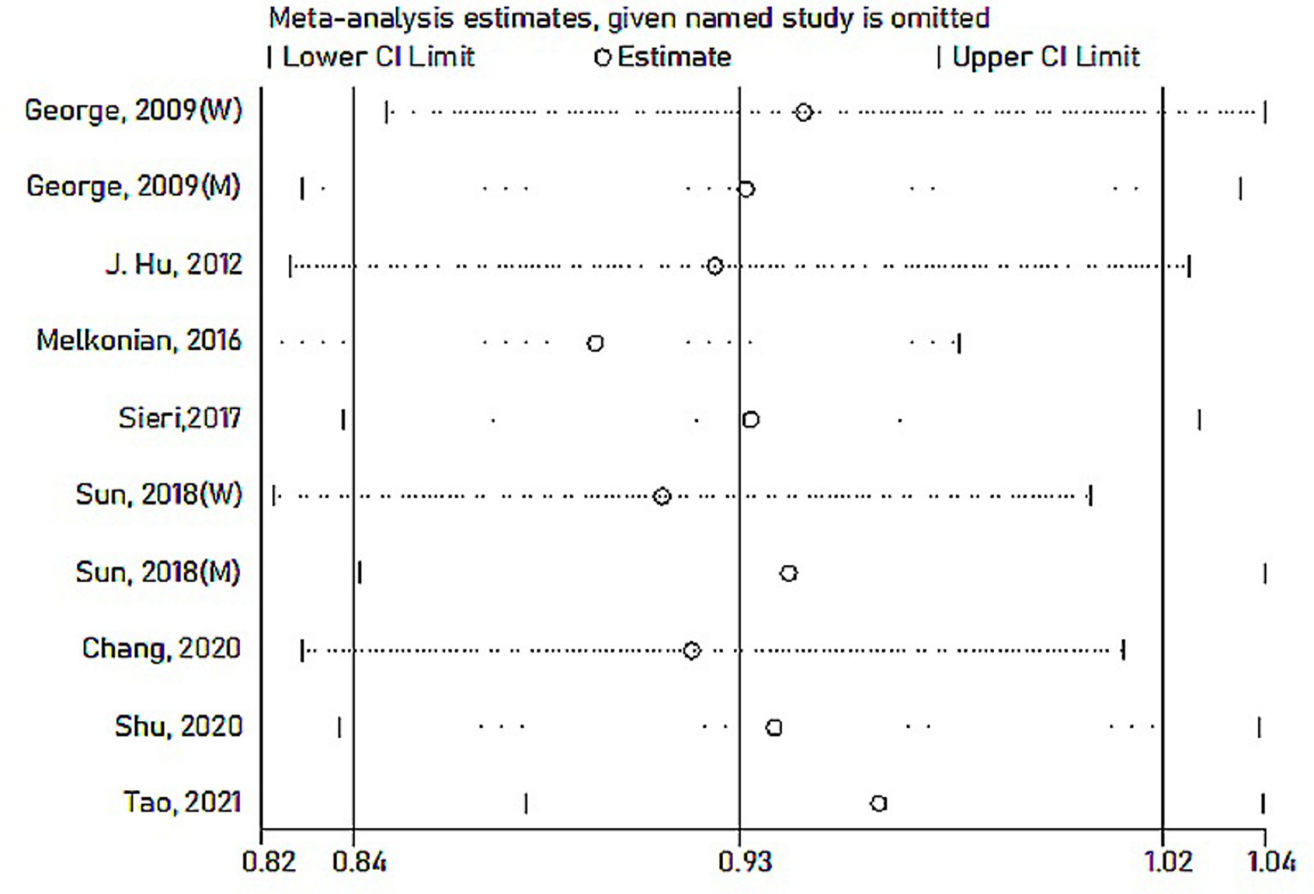
Sensitivity analysis for the association between GL and lung cancer.

The multivariable-adjusted RRs of GL and lung cancer risk are shown in Fig 6. These results were different between GL and lung cancer risk from 10 studies. The combined RR was 0.93 (95% CI: 0.84–1.02), without evident heterogeneity (*P* = 0.076, *I*^*2*^ = 42.3%). The funnel plot, Egger’s test (*P* = 0.400), and Begg’s test (*P* = 0.721) showed no publication bias from the included studies. The funnel plot of GL is presented in Fig 7. A study showed that the overall estimates ranging from 0.89 (95% CI: 0.83–0.96) to 0.92 (95% CI: 0.89–1.03) were substantially affected. The pooled results remained unchanged in this sensitivity analysis (Fig 8). However, the results were inconsistent when we stratified by design (Table 3).

## Discussion

GI and GL can affect lung cancer development, but with inconsistent results [38]. Turati et al. reported a non-significant meta-analysis, and sources of heterogeneity were not examined [22]. Another meta-analysis was only performed for cohort studies [21]. As the available evidence has increased and more studies have been carried out, we have increased our statistical power to detect any association between GI or GL and lung cancer risk. In this meta-analysis, these findings showed that GI was associated with the risk of lung cancer. The association between GI and lung cancer risk did not remarkably change when stratified by study design, gender, geographic area, quality, and adjustment for energy intake. This trend was also seen in other types of cancer [15, 39]. However, the GI is not consistent in various human malignancies [40]. The possible mechanism included chronic hyperinsulinemia and insulin resistance. A high GI diet can provoke postprandial glycemic spikes, and insulin resistance may frequently be accompanied by hyperinsulinemia [41]. Insulin can promote cell growth, mitosis, and migration and prevent cell apoptosis, \ an important growth factor [42]. In addition, insulin can stimulate the farnesylated Ras protein on the plasma membrane, regulating the cell’s response to other growth factors and enhancing their mitogenic effects [43]. A high GI diet requires insulin, and the insulin–IGF axis has been associated with increased cancer risk [44]. Biological activity IGF-1 is involved in cancer progression by preventing cell apoptosis and facilitating cell proliferation after binding to the IGF-1 receptor [45]. Furthermore, substantial heterogeneity across studies on the relationships of GI with lung cancer risk significant heterogeneity was observed. However, no covariate significantly contributed to heterogeneity. This situation was common when the study designs were diverse and the population characteristics among the included studies were inconsistent. Moreover, detailed information on cancer staging and severity should be included to explore this association.

GL is the product of carbohydrate intake in each serving of food and GI, which better presented the importance of the quantity of carbohydrates [7]. In our analysis, we found that dietary GL was not associated with lung cancer risk, consistent with a previous meta-analyses [22]. Dietary GL was primarily associated with the intake of carbohydrate-rich foods, suggesting that it may be closely related to total carbohydrate intake. However, sensitivity analysis reveals one negative correlation between GL and lung cancer risk, which significantly affected the combined results. Thus, the result must be carefully interpreted. In addition, Tao explained the GL effects and presented the overall benefits of carbohydrates for preventing lung cancer development [37]. In George et al.’s study among women, a high dietary GL was also associated with a reduced risk of pancreatic cancer (RR = 0.49; *P* for trend = 0.04), and the GL association may also be aroused by residual confounding or methodological heterogeneity among the studies [29]. The relationship between GL and the risk of lung cancer was also inconsistent in our case-control and cohort studies. Several reasons may explain the difference between case-control studies and prospective cohort studies. First, case-controlled studies might be affected by certain biases, such as the recall and selection, particularly dietary recall bias. The second reasons of the inconsistent results may be attributable to the small number of cases-control studies. For a comprehensive understanding of the results of our case-control studies, only three studies were analyzed, and no unambiguous result of an association between GL and lung cancer risk was observed (RR = 1.08, 95% CI: 0.94–1.23). In addition, a strong negative correlation between GL and lung cancer risk was found (RR = 0.87, 95% CI: 0.80–0.94) in a systematic meta-analysis of seven cohort studies, which agreed with those obtained by Long et al. [21]. Therefore, more evidences are required to clearly understand the relationship between GL and the risk of lung cancer.

Our analysis has several strengths. First, a thorough literature search was performed, and no evident publication bias was found by statistical methods. Second, using available evidence and large sample size with a long duration involved (1998 to 2021), thereby confirming the reliable results for the association between GI or GL and lung cancer risk. Moreover, in the study quality assessment, most reports scored above 6, providing a good basis for this research.

Our study involved several limitations. First, a marked difference was observed in leave-one-out sensitivity analyses of GL, indicating that the pooled risk estimates were influenced by one study. Second, using an FFQ to evaluate GI and GL may have generated measurement errors for other studies. GI or GL was calculated based on a limited number of food items in most dietary questionnaires, which might have influenced the accuracy of the exposure assessment. To achieve more precise and convincing evidence, a well-designed method to evaluate GI or GL is required. Third, all risk estimates were calculated from multivariable models, but potential risk factors were not adjusted consistently by individual studies. Considerable research on risk factors and earlier studies fail to adjust for potential confounders; thus, the results may be affected by other confounding factors. Therefore, prospective studies and other large-scale multi-center data are necessary to substantiate these findings. Finally, the number of the included studies in the dose-response analyses was relatively limited, the results should be considered with cautions.

In summary, there was a positive correlation between GI and lung cancer risk, but no significant correlation was observed between GL and lung cancer risk. The present meta-analysis provides evidence to disclose the intrinsic link between GI or GL and lung cancer, although dose-response meta-analyses are still required. Exploring the mechanisms linking GI or GL, the IGF axis, and lung cancer risk further in humans is important and may determine the association between diet and lung cancer.

## Supporting information

S1 Checklist PRISMA checklist. (DOC)

S1 Dataset (DOC)
