# Supplementary material for: Glycemic index, glycemic load, and lung cancer risk: A meta-analysis of cohort and case-control studies
Source: PLoS One. 2022 Sep 1;17(9):e0273943. doi: 10.1371/journal.pone.0273943 (PMC9436153; doi:10.1371/journal.pone.0273943)
Supplement: S1 Dataset — (DOC) [file pone.0273943.s002.doc]

**Table 1 Details of data included in the meta analysis**

| **AUTHOR AND YEAR OF PUBLICATION** | **THEME** | **CRITERIA** | **RR** | **LCI** | **UCI** |
| --- | --- | --- | --- | --- | --- |
| De Stefani, 1998 | GI and Lung cancer | Case control study | 2.77 | 1.28 | 5.97 |
| George, 2009(W) | GI and Lung cancer | Cohort Study | 1.12 | 0.98 | 1.27 |
| George, 2009(M) | GI and Lung cancer | Cohort Study | 1.16 | 0.92 | 1.47 |
| J. Hu, 2012 | GI and Lung cancer | Case control study | 1.04 | 0.89 | 1.23 |
| Melkonian, 2016 | GI and Lung cancer | Case control study | 1.48 | 1.21 | 1.83 |
| Sieri,2017 | GI and Lung cancer | Cohort Study | 0.88 | 0.53 | 1.46 |
| Sun, 2018(W) | GI and Lung cancer | Cohort Study | 1.06 | 0.86 | 1.3 |
| Sun, 2018(M) | GI and Lung cancer | Cohort Study | 0.83 | 0.67 | 1.03 |
| Chang, 2020 | GI and Lung cancer | Case control study | 1.62 | 1.17 | 2.25 |
| Shu, 2020 | GI and Lung cancer | Cohort Study | 1.06 | 0.86 | 1.3 |
| Tao, 2021 | GI and Lung cancer | Cohort study | 1.19 | 1.05 | 1.35 |
| George, 2009(W) | GL and Lung cancer | Cohort Study | 0.81 | 0.64 | 1.03 |
| George, 2009(M) | GL and Lung cancer | Cohort Study | 0.93 | 0.78 | 1.11 |
| J. Hu, 2012 | GL and Lung cancer | Case control study | 0.98 | 0.8 | 1.21 |
| Melkonian, 2016 | GL and Lung cancer | Case control study | 1.16 | 0.94 | 1.42 |
| Sieri,2017 | GL and Lung cancer | Cohort Study | 0.88 | 0.53 | 1.46 |
| Sun, 2018(W) | GL and Lung cancer | Cohort Study | 1.09 | 0.86 | 1.37 |
| Sun, 2018(M) | GL and Lung cancer | Cohort Study | 0.85 | 0.68 | 1.05 |
| Chang, 2020 | GL and Lung cancer | Case control study | 1.13 | 0.79 | 1.64 |
| Shu, 2020 | GL and Lung cancer | Cohort Study | 0.88 | 0.71 | 1.07 |
| Tao, 2021 | GL and Lung cancer | Cohort Study | 0.76 | 0.65 | 0.9 |

**Table 2 Details of data included in the dose response meta-analysis of GI**

| **AUTHOR AND YEAR OF PUBLICATION** | **TYPE** | **DOSE** | **CASES** | **N** | **RR** | **LCI** | **UCI** |
| --- | --- | --- | --- | --- | --- | --- | --- |
| George, 2009(W) | Cohort Study | 0 | 2288 | 183,535 | 1 | 1 | 1 |
| George, 2009(W) | Cohort Study | 9.48 | 2288 | 183,535 | 1.07 | 0.93 | 1.22 |
| George, 2009(W) | Cohort Study | 11.46 | 2288 | 183,535 | 1.01 | 0.88 | 1.16 |
| George, 2009(W) | Cohort Study | 13.455 | 2288 | 183,535 | 0.98 | 0.86 | 1.13 |
| George, 2009(W) | Cohort Study | 28.23 | 2288 | 183,535 | 1.12 | 0.98 | 1.27 |
| George, 2009(M) | Cohort Study | 0 | 3769 | 262,642 | 1 | 1 | 1 |
| George, 2009(M) | Cohort Study | 9.91 | 3769 | 262,642 | 1.01 | 0.89 | 1.11 |
| George, 2009(M) | Cohort Study | 11.8 | 3769 | 262,642 | 1.04 | 0.93 | 1.16 |
| George, 2009(M) | Cohort Study | 13.645 | 3769 | 262,642 | 1.01 | 0.9 | 1.11 |
| George, 2009(M) | Cohort Study | 28.19 | 3769 | 262,642 | 1.08 | 0.98 | 1.2 |
| J. Hu, 2012 | Case control study | 0 | 3341 | 8380 | 1 | 1 | 1 |
| J. Hu, 2012 | Case control study | 27.952 | 3341 | 8380 | 1.27 | 1.07 | 1.5 |
| J. Hu, 2012 | Case control study | 31.912 | 3341 | 8380 | 1.1 | 0.94 | 1.3 |
| J. Hu, 2012 | Case control study | 76.937 | 3341 | 8380 | 1.04 | 0.89 | 1.23 |
| Sun, 2018(W) | Cohort Study | 0 | 128 | 266032 | 1 | 1 | 1 |
| Sun, 2018(W) | Cohort Study | 5.84 | 157 | 266779 | 1.15 | 0.91 | 1.46 |
| Sun, 2018(W) | Cohort Study | 9.14 | 164 | 266732 | 1.12 | 0.89 | 1.42 |
| Sun, 2018(W) | Cohort Study | 13.11 | 200 | 262379 | 1.16 | 0.92 | 1.47 |
| Sun, 2018(M) | Cohort Study | 0 | 182 | 138319 | 1 | 1 | 1 |
| Sun, 2018(M) | Cohort Study | 5.87 | 139 | 138152 | 0.81 | 0.65 | 1.01 |
| Sun, 2018(M) | Cohort Study | 9.11 | 178 | 137398 | 0.99 | 0.81 | 1.23 |
| Sun, 2018(M) | Cohort Study | 12.89 | 164 | 136912 | 0.83 | 0.67 | 1.03 |
| Chang, 2020 | Case control study | 0 | 142 | 483 | 1 | 1 | 1 |
| Chang, 2020 | Case control study | 16.035 | 185 | 528 | 1.29 | 0.93 | 1.77 |
| Chang, 2020 | Case control study | 24.635 | 266 | 608 | 1.62 | 1.17 | 2.25 |
| Shu, 2020 | Cohort Study | 0 | 166 | 88298 | 1 | 1 | 1 |
| Shu, 2020 | Cohort Study | 2.4 | 190 | 87,982 | 1.14 | 0.92 | 1.4 |
| Shu, 2020 | Cohort Study | 3.8 | 185 | 88,303 | 1.04 | 0.84 | 1.28 |
| Shu, 2020 | Cohort Study | 5.2 | 202 | 87,484 | 1.11 | 0.91 | 1.37 |
| Shu, 2020 | Cohort Study | 7.6 | 204 | 87,430 | 1.06 | 0.86 | 1.3 |
| Tao, 2021 | Cohort Study | 0 | 2094 | 113096 | 1 | 1 | 1 |
| Tao, 2021 | Cohort Study | 3.4 | 2094 | 113096 | 0.99 | 0.87 | 1.13 |
| Tao, 2021 | Cohort Study | 5.4 | 2094 | 113096 | 1.06 | 0.93 | 1.21 |
| Tao, 2021 | Cohort Study | 8.4 | 2094 | 113096 | 1.19 | 1.05 | 1.35 |
